# Sequential infection of *Daphnia magna* by a gut microsporidium followed by a haemolymph yeast decreases transmission of both parasites

**DOI:** 10.1017/S0031182021001384

**Published:** 2021-11

**Authors:** Florent Manzi, Snir Halle, Louise Seemann, Frida Ben-Ami, Justyna Wolinska

**Affiliations:** 1 Department of Ecosystem Research, Leibniz-Institute of Freshwater Ecology and Inland Fisheries, Berlin, Germany; 2 Department of Biology, Chemistry, Pharmacy, Institute of Biology, Freie Universität Berlin, Berlin, Germany; 3 School of Zoology, George S. Wise Faculty of Life Sciences, Tel Aviv University, Tel Aviv 6997801, Israel

**Keywords:** Coinfections, *Metschnikowia*, *Ordospora*, priority effects, zooplankton

## Abstract

Over the course of seasonal epidemics, populations of susceptible hosts may encounter a wide variety of parasites. Parasite phenology affects the order in which these species encounter their hosts, leading to sequential infections, with potentially strong effects on within-host growth and host population dynamics. Here, the cladoceran *Daphnia magna* was exposed sequentially to a haemolymph-infecting yeast (*Metschnikowia bicuspidata*) and a gut microsporidium (*Ordospora colligata*), with experimental treatments reflecting two possible scenarios of parasite succession. The effects of single and co-exposure were compared on parasite infectivity, spore production and the overall virulence experienced by the host. We show that neither parasite benefited from coinfection; instead, when hosts encountered *Ordospora*, followed by *Metschnikowia*, higher levels of host mortality contributed to an overall decrease in the transmission of both parasites. These results showcase an example of sequential infections generating unilateral priority effects, in which antagonistic interactions between parasites can alleviate the intensity of infection and coincide with maladaptive levels of damage inflicted on the host.

## Graphical abstract

**Figure:**
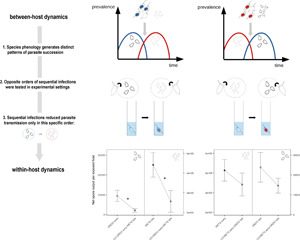

## Introduction

Over the course of their lifetime, most free-living organisms are bound to encounter parasites (Poulin and Morand, 2000). Realistically, individual hosts rarely encounter a single parasite, but rather progress through a series of events (exposure, infection and recovery) from a multitude of pathogens, some of which may coexist within the course of an infection. While some parasites may encounter their hosts simultaneously, such as several virus species being inoculated by a shared vector (Swanson *et al*., 2006), the majority of multiple infections are thought to occur sequentially (Karvonen *et al*., 2019). In a within-host framework, ‘priority effects’ occur when this sequence of infection alters the outcome of interactions among parasites (Halliday *et al*., 2020). For instance, as different strains compete for a pool of susceptible hosts, faster replicating strains are generally favoured (Levin and Pimentel, 1981; Nowak and May, 1994). However, prior residency may allow ‘weaker’ strains to prevail in coinfection, by conferring protection against more competitive genotypes (Ben-Ami *et al*., 2008; Seifi *et al*., 2012). The biological mechanisms underlying such observations are likely the product of complex interactions between the defending host and coinfecting parasites (Alizon *et al*., 2013), although common hypotheses have been proposed, which generally involve host immunity and competition for resources (Read and Taylor, 2001; de Roode *et al*., 2005). For instance, prior exposure may weaken host immunity in such a way that secondary infections are facilitated (Graham, 2008) or trigger priming of the host's defences, so that subsequent infections are either alleviated (Rodrigues *et al*., 2010) or prevented altogether (Ratcliff *et al*., 1999; Syller and Grupa, 2016). Prior infection can also sequester within-host resources, which will then alter the developmental trajectory of late-arriving parasites (Graham, 2008). Although traditionally used in the context of species assemblages and community structures (Connell and Slatyer, 1977; Wilbur and Alford, 1985), this notion of priority effects has since been widely applied to the study of sequential infections (Hoverman *et al*., 2013; Wuerthner *et al*., 2017; Clay *et al*., 2018; Carpenter *et al*., 2021). Incidentally, a majority of studies have reported negative effects on later arriving parasites (reviewed in Karvonen *et al*., 2019; but see also Ezenwa *et al*., 2010, Lohr *et al*., 2010*b*).

Over the past decade, water fleas of the genus *Daphnia* (Crustacea: Cladocera) and their microparasites have emerged as an ecologically relevant system for testing the outcome of interspecific coinfections (Ben-Ami *et al*., 2011; Lange *et al*., 2014; Sánchez *et al*., 2019). As common inhabitants and crucial agents in the stability of freshwater ecosystems (Carpenter *et al*., 1985; Lampert, 2006, 2011), *Daphnia* are known to harbour a functionally and taxonomically diverse range of parasite species, including microsporidia, fungi, ichthyosporea, bacteria (Ebert, 1995; Stirnadel and Ebert, 1997; Wolinska, *et al*., 2009; Goren and Ben-Ami, 2013) and viruses (Toenshoff *et al*., 2018). For example, the gut microsporidium *Ordospora colligata* (Microsporidia: Ordosporidae, hereafter referred to as *Ordospora*) can be found in northern and western European ponds (Ebert, 2005), where high prevalences have been recorded in populations of its only host, *Daphnia magna* (Ebert *et al*., 2001; Decaestecker *et al*., 2005). In temperate ponds, the prevalence of microsporidian parasites increases from late spring to early summer, before waning back in the autumn and winter (Ebert, 1995; Larsson *et al*., 1997). Epidemics usually start from infectious spore banks contained in the sediment, although transmission stages are also able to disperse in the water, where they can be encountered as free-floating spores (Mangin *et al*., 1995; Kirk *et al*., 2018). Microsporidian spores exhibit high survivability outside their hosts, allowing the parasite to overwinter and survive periods of host diapause (Ebert, 1995). Another common parasite of *Daphnia*, the waterborne yeast *Metschnikowia bicuspidata* (Ascomycota: Saccharomycetales, hereafter referred to as *Metschnikowia*) is a generalist capable of infecting several zooplankton species (Ebert, 2005; Dallas *et al*., 2016). In temperate freshwater bodies of the Northern Hemisphere, epidemics of *Metschnikowia* typically peak in the late summer to early autumn (Duffy *et al*., 2009; Hall *et al*., 2011; Penczykowski *et al*., 2014), although it has been found to overlap with gut microsporidia in the summer period (Ebert, 1995; Stirnadel and Ebert, 1997) or during the rainy season in Mediterranean to semi-arid climates of the Middle East (Goren and Ben-Ami, 2013). Transmission is also horizontal, although infective propagules are only released from dead hosts (i.e. obligate killer), and thus mostly restricted to the sediment (Duffy, 2009; Duffy and Hunsberger, 2019).

Due to their overlapping distribution, coinfections of *D. magna* involving both taxa are likely to occur. However, these phylogenetically distant species have been shown to differ greatly in their overall reproductive strategy: while infections by *Ordospora* typically reduce host lifespan by up to 20% (Ebert *et al*., 2000), *Metschnikowia* is a highly virulent parasite, producing lethal infections under 2-to-3 weeks (Ebert, 2005). Because virulence in coinfection generally aligns with the amount of damage induced by the more virulent parasite (Ben-Ami *et al*., 2008; Ben-Ami and Routtu, 2013), coinfection by an obligate killer may drastically reduce the timespan available to efficiently exploit host resources for growth (Lohr *et al*., 2010*b*; Clay *et al*., 2019). Furthermore, within-host competition for resources may be particularly relevant for parasites that colonize distinct niches within the host (Ben-Ami *et al*., 2011). The intracellular *Ordospora* ensures reproduction by hijacking energy (i.e. ATP molecules) within the cytoplasm of epithelial cells (Tsaousis *et al*., 2008), which serves both as a barrier and interface between the gut lumen and the haemolymph. Meanwhile, development of *Metschnikowia* takes place in the body cavity (Codreanu and Codreanu-Balcescu, 1981), which is in turn alimented by direct trophic exchanges along these compartments.

In addition to their contrasting reproductive strategies, the exact sequence in which parasites succeed each other within one host may further complicate such interactions (Hood, 2003; de Roode *et al*., 2005; Jäger and Schjørring, 2006). The documented phenology of both parasites suggests that infections are likely to overlap in late summer, with a predicted prior presence of *Ordospora* in sympatric populations. Incidentally, some studies of priority effects have been conducted using *Metschnikowia*, along with ichtyhosporean (Lohr *et al*., 2010*b*) and bacterial (Clay *et al*., 2019) parasites of *Daphnia*, in which it was shown to consistently experience impaired transmission under prior residency. However, the literature is currently lacking such experimental assays for microsporidian parasites of *Daphnia*. In their exploratory study, Mangin *et al*. (1995) reported successful transmission of *Ordospora* to individuals previously infected with the microsporidium *Tuzetia* sp. (now referred to as *Hamiltosporidium magnivora*, Haag *et al*., 2011). Nevertheless, systematic assays of sequential exposure using *Ordospora* have not been documented.

Here, we sequentially exposed the host *D. magna* to the parasites *Metschnikowia* and *Ordospora*. Experimental treatments were designed to reflect two possible scenarios of parasite succession: one in which a gut microsporidium (*Ordospora*) encounters the host after prior establishment of a fungal parasite in the haemolymph (*Metschnikowia*), and a second, opposite scenario in which the haemolymph-infecting yeast encounters the host after prior establishment of the gut parasite. We aimed to determine (i) whether sequential infections differ from single infections in terms of parasite transmission traits, specifically addressing the following questions: (a) how does *Metschnikowia* respond to later arrival of *Ordospora*; (b) how does *Metschnikowia* respond to prior infection by *Ordospora*; (c) how does *Ordospora* respond to later arrival of *Metschnikowia* and (d) how does *Ordospora* respond to prior infection by *Metschnikowia*; and (ii) whether opposite scenarios of parasite succession influence host fitness in diverging ways.

## Materials and methods

### Study system

*Daphnia magna* is commonly found in lakes and temporary freshwater bodies of the Northern Hemisphere (Ebert, 2005). Due to its large size (i.e. up to 5 mm) and efficient filtering rate, *D. magna* is particularly prone to multiple infections in general, as compared with smaller sympatric species (Stirnadel and Ebert, 1997). One clonal line of *D. magna* was used as the focal host for this experiment (clone NO-V-7, isolated from Norway; Haag *et al*., 2020). This single genotype was selected on the basis of having the highest compatibility with both strains of parasites used in this study, as reported by preliminary infectivity assays.

A single strain of the yeast *Metschnikowia* was used, isolated from Ammersee, Germany and later propagated on lab-reared *D. magna* (clone E17:07). Spores are needle-shaped and puncture the gut epithelium to reach the haemolymph, where fungal development takes place (Codreanu and Codreanu-Balcescu, 1981; Stewart Merrill and Cáceres, 2018). Infection symptoms are clearly visible after 9–10 days, when the host's body cavity starts to fill with elongated asci (Stewart Merrill and Cáceres, 2018).

A single strain of *Ordospora* was used, isolated and maintained on lab-reared cultures of the experimental host (NO-V-7). Late stages of infection are characterized by the presence of several dozens of spore clusters in the gut epithelium, which are mostly confined to the upper half of the gut epithelium (Refardt and Ebert, 2006) and notably visible in the ‘angular’ sections of the digestive tract, such as the anterior diverticuli (Ebert, 2005). Spore release can occur from live host after 3 days (Mangin *et al*., 1995; Refardt and Ebert, 2007), although reliable detection of infection is usually possible after 11 days, due to the exponential increase in parasite spore load throughout the infection (Kirk *et al*., 2019).

### Experimental setup

**Fig. 1.**
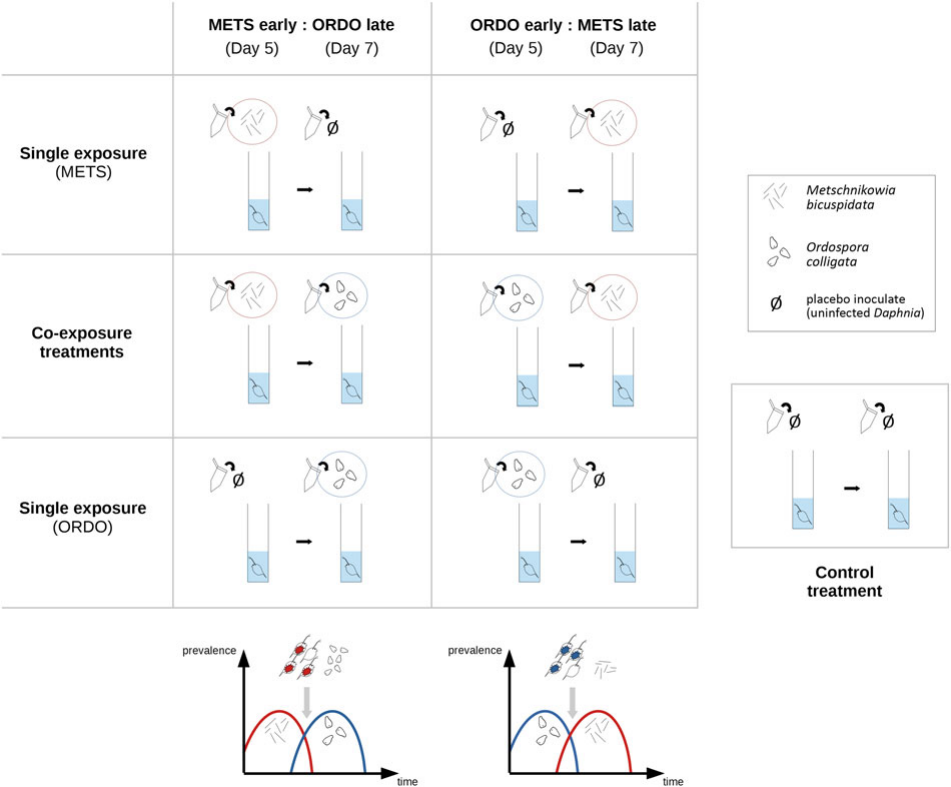
Graphical representation of the six exposure treatments, corresponding to two possible scenarios of parasite succession. On the left, the haemolymph parasite *Metschnikowia bicuspidata* arrives ‘early’ and the gut parasite *Ordospora colligata* arrives ‘late’. On the right, the gut parasite *O. colligata* arrives ‘early’ and the haemolymph parasite *M. bicuspidata* arrives ‘late’. Single-exposure treatments within each scenario follow the same timing of infection as the co-exposure treatment, to allow proper comparison of parasite and host fitness parameters across single and co-exposure settings. The control treatment received the same placebo inoculate (obtained from crushed uninfected *Daphnia*) as single-exposure treatments, albeit on both inoculation days.

The experimental design included four single-exposure treatments (‘METS early’: exposed to spores of *Metschnikowia* on day 5; ‘METS late’: exposed to spores of *Metschnikowia* on day 7; ‘ORDO early’: exposed to spores of *Ordospora* on day 5; ‘ORDO late’: exposed to spores of *Ordospora* on day 7), two co-exposure treatments (‘CO:METS early:ORDO late’: exposed to spores of *Metschnikowia* on day 5 and *Ordospora* on day 7; ‘CO:ORDO early:METS late’: exposed to spores of *Ordospora* on day 5 and *Metschnikowia* on day 7) and one control treatment (exposed to crushed tissue of uninfected *D. magna* on both days). On the day which did not feature exposure to the parasite, all single infection treatments were exposed to the same placebo as the control. Forty replicates (individual *Daphnia*) were used for each treatment, yielding a total of 280 experimental units (Fig. 1).

### Inoculation process

Juvenile *Daphnia* born within a 24-h time span (i.e. day 1) were transferred to individual jars containing 5 mL of synthetic culture medium (SSS-medium, Saebelfeld *et al*., 2017). *Daphnia* were maintained at a constant temperature of 19°C, under a 12:12 light–dark photoperiod and fed three times per week with 1 mg C L^−1^ of *Scenedesmus obliquus* (green algae, maintained in WC algal medium). On day 5, spore solutions were prepared for both parasites. Infected individuals were gathered in Eppendorf tubes and crushed with a plastic pestle. The equivalent of ten adult *Daphnia* were crushed per 40 replicates, ensuring a balanced amount of host tissue was introduced in all seven treatments. To prepare the stock solution of *Metschnikowia*, the appropriate number of infected *Daphnia* (clone E17:07) was crushed to achieve a target dose of 17 500 spore per recipient *Daphnia* (3500 spores mL^−1^) across 80 replicates in two treatments (METS early; CO:METS early:ORDO late). This dose was comparably higher than previous studies utilizing the same system (Hesse *et al*., 2012), in order to maximize chances of successful coinfection in the co-exposure treatments. The solution was completed by crushing additional uninfected individuals up to a total of 20. To prepare the stock solution of *Ordospora*, 20 *Daphnia* (clone NO-V-7) presenting signs of late stage infection (large amount of spore clusters in the gut) were crushed to achieve a target dose of approximately 38 000 spores per recipient *Daphnia* (7600 spores mL^−1^) across 80 replicates in two treatments (ORDO early; CO:ORDO early:METS late). Repeated counts from stock cultures were shown to provide the required number of spores from 20 individuals (CI_95%_ of average spore yield per inoculation dose: 38 100 ± 6.5%). These spore solutions were then distributed across all replicates of their respective treatments. Single infection treatments that did not receive spores on day 5 (METS late; ORDO late), as well as the control treatment were exposed to a placebo inoculate, prepared by crushing uninfected individuals (clone NO-V-7) using the same ratio of ten adult *Daphnia* for each treatment of 40 replicates.

After an exposure period of 2 days allocated to the first parasite, all *Daphnia* were transferred to 5 mL of clean medium, and the inoculation process was repeated on day 7. This delay was chosen to ensure that either *Metschnikowia* (Stewart Merrill and Cáceres, 2018) or *Ordospora* (Mangin *et al*., 1995; Refardt and Ebert, 2007) would reach their target compartment, before exposing the host to the second parasite (consistent with the definition of sequential infection as following establishment of the prior parasite; Marchetto and Power, 2018). Spore solutions were prepared anew, using the same methods as described for day 5, and inoculated into their respective treatments (METS late; CO:ORDO early:METS late; ORDO late; CO:METS early:ORDO late). *Daphnia* were not fed on either exposure day, in order to promote spore uptake (Hall *et al*., 2007). On experimental day 9 (i.e. the end of the exposure period allocated to the second parasite), *Daphnia* were transferred to 20 mL of fresh, spore-free medium.

From day 9 onwards (both exposure periods having been completed), dead individuals were collected and fixed in 3.7% formaldehyde. Samples were kept at 4°C until the assessment of spore production (see below). Juveniles were removed and counted daily, and *Daphnia* were transferred to fresh medium (20 mL) every 4 days. The experiment was terminated on day 81, when the last surviving *Daphnia* in the control treatment had died.

### Recorded parameters

#### Parasite fitness

Individual *Daphnia* from all treatments were assigned a binary value for host viability (0 = early death, 1 = viable host). Viable hosts were described as individual *Daphnia* having survived until the first possible detection of infection symptoms (i.e. presence of spores from crushed individuals), which was determined as 9 days post-exposure for *Metschnikowia* (Stewart Merrill and Cáceres, 2018) and 11 days post-exposure for *Ordospora* (Kirk *et al*., 2019). Individuals from the six exposure treatments were assigned a separate value for parasite infectivity (0 = non infected, 1 = infected). Infected hosts were described as individual *Daphnia* in which spores of either parasite were detected (among those considered viable). Individuals which did not survive until at least both inoculation events had occurred (i.e. beyond experimental day 7) were excluded from both calculations, as these could not be properly attributed to their intended treatments (Appendix, Table S1). All retrieved samples (except for the control) were blinded to ensure reliable assessment of spore yield upon host death across single and co-exposure treatments. Samples were crushed in 0.3 mL, homogenized and loaded with 10 *μ*L in a Neubauer Improved chamber. Samples were first screened for detection and quantification of needle-shaped *Metschnikowia* spores, under a Nikon SMZ25 stereomicroscope (200× magnification). For identification and quantification of *Ordospora*, samples were observed under a Nikon Ti Eclypse inverted microscope, using phase contrast and UV exposure (200× magnification); for each sample, 2 *μ*L of Calcofluor-White (1 mg mL^−1^) were added to the counting chamber to generate blue fluorescence, thereby staining the chitin-rich wall of pyriform spores (Krebs *et al*., 2017).

Parasite growth (i.e. the rate of spore production) was computed as the ratio of spore yield over the number of days survived by the host post-exposure. A comprehensive measure of parasite fitness, the net spore output per exposed host, was computed as an estimation of overall transmission success. Here, in addition to individuals that produced a detectable spore yield, those that scored ‘0’ for either host viability or parasite infectivity were also included, and recorded as a having a ‘net’ spore output of zero. This was done to reflect the probability of each encounter with an exposed host leading to subsequent reproduction of the parasite, which may differ across experimental treatments, independently of parasite growth (Manzi *et al*., 2020).

#### Host fitness

Host fitness was recorded *via* three variables: host lifespan post-exposure was defined as the number of days survived by individual *Daphnia*, following the completion of both exposure events (i.e. beyond experimental day 7). Total offspring production per individual was used as a comprehensive measure of the host's reproductive success. Finally, the rate of offspring production was computed as the ratio of total offspring production over host lifespan post-exposure.

### Data analysis

Data were analysed using R version 4.0.4 (R Core Team, 2021). Graphical outputs were produced using the ‘ggplot2’ (Wickham, 2016), ‘Hmisc’ (Harrell and Harrell, 2019) and ‘patchwork’ (Pedersen, 2020) packages. Analyses of variance (*F*-test or *χ*^2^ test) were performed with the ‘car’ package (Fox and Weisberg, 2019).

#### Parasite fitness

Parasite fitness variables were analysed separately for each parasite and compared across single and co-exposure treatments with the same timing of infection. Host viability (0 = early death, 1 = viable host) and parasite infectivity (0 = non infected, 1 = infected) were analysed using a binary logistic regression with ‘exposure’ as explanatory variable (i.e. a factor with up to six possible levels). Additionally, host viability was compared to baseline mortality in the control treatment (Appendix, Table S2). In co-exposure treatments, infectivity of a given parasite included the total number of cases in which spores of that parasite were detected, either in single or coinfection. Parasite growth and the net spore output per exposed host were analysed with ‘exposure’ as explanatory variable in a linear model, assuming a normal distribution of residuals. Only successful infections (i.e. detection of a non-zero number of spores) were included in the analysis of parasite growth. All individuals which survived until at least both exposure events had occurred (i.e. beyond experimental day 7) were included in the analysis of net spore output. Normal distribution and homoscedasticity of the residuals were verified by visual inspection of quantile–quantile plots and residuals against fitted values.

#### Host fitness

Host fitness variables (namely lifespan post-exposure, rate of offspring production and total offspring production) were analysed using linear models, assuming a normal distribution of residuals, with ‘exposure’ as the explanatory variable (i.e. a factor with seven levels, including the control treatment). Only individuals successfully infected by either one (single exposure) or both parasites (co-exposure) were included in the non-control treatments. One individual from the control treatment was lost due to handling error and was thus excluded from these analyses. Post-hoc pairwise comparisons (Tukey's HSD test) were performed with the ‘multcomp’ package (Hothorn *et al*., 2008).

## Results

### Parasite fitness

#### How does *Metschnikowia* respond to later arrival of *Ordospora*?

**Fig. 2.**
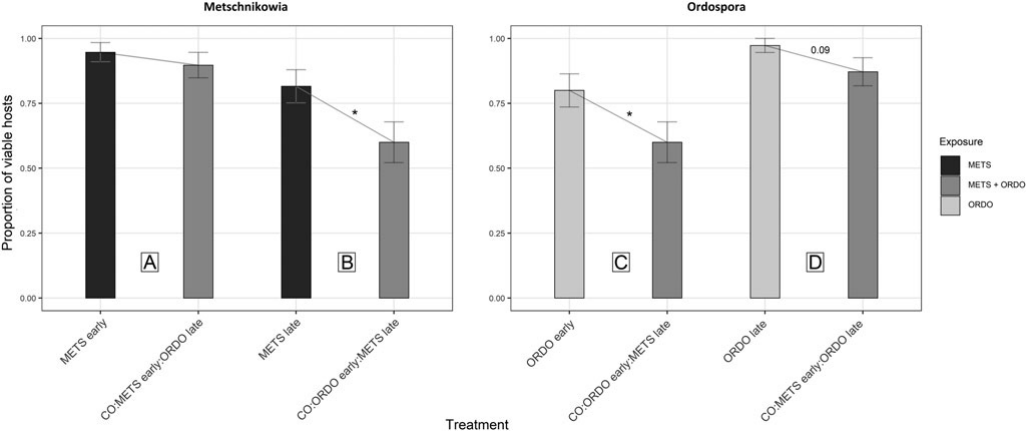
Graphical representation of the proportion of *Daphnia* considered viable hosts, i.e. which survived until at least 9 days post-exposure (*Metschnikowia*) or 11 days post-exposure (*Ordospora*), allowing either parasite to produce detectable levels of infection (i.e. presence of spores in crushed individuals). Host viability was compared between single and co-exposure treatments, to answer the following: (A) How does *Metschnikowia* respond to later arrival of *Ordospora*? (B) How does *Metschnikowia* respond to prior infection by *Ordospora*? (C) How does *Ordospora* respond to later arrival of *Metschnikowia*? (D) How does *Ordospora* respond to prior infection by *Metschnikowia*? Individuals which did not survive until at least both inoculation events had occurred were excluded from these calculations. Error bars depict the standard error of the mean (calculated from binary values assigned to individual *Daphnia*: 0 = early death, 1 = viable host). Significance levels are provided by logistic regression performed across single and co-exposure treatments with shared timing of infection: *P ⩽ 0.05.

**Fig. 3.**
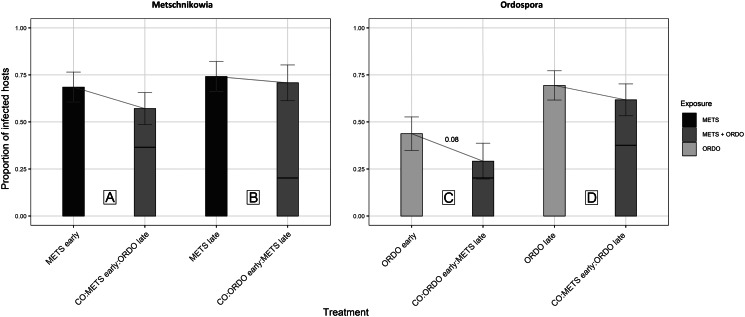
Graphical representation of the proportion of *Daphnia* successfully infected by the parasites *Metschnikowia* and *Ordospora*. Parasite infectivity was compared between single and co-exposure treatments, to answer the following: (A) How does *Metschnikowia* respond to later arrival of *Ordospora*? (B) How does *Metschnikowia* respond to prior infection by *Ordospora*? (C) How does *Ordospora* respond to later arrival of *Metschnikowia*? (D) How does *Ordospora* respond to prior infection by *Metschnikowia*? The horizontal section of the bar in co-exposure treatments represents the contribution of coinfections to the overall number of successful infections by the focal parasite. Individuals which did not survive until the earliest possible observation of parasite symptoms were excluded from the analysis of infectivity; reported proportions are computed amongst the remaining number of individuals considered viable. Error bars depict the standard error of the mean (calculated from binary values assigned to individual *Daphnia*: 0 = non infected, 1 = infected). Significance levels are provided by logistic regression performed across single and co-exposure treatments with shared timing of infection; none of the pairwise comparisons were significant.

**Fig. 4.**
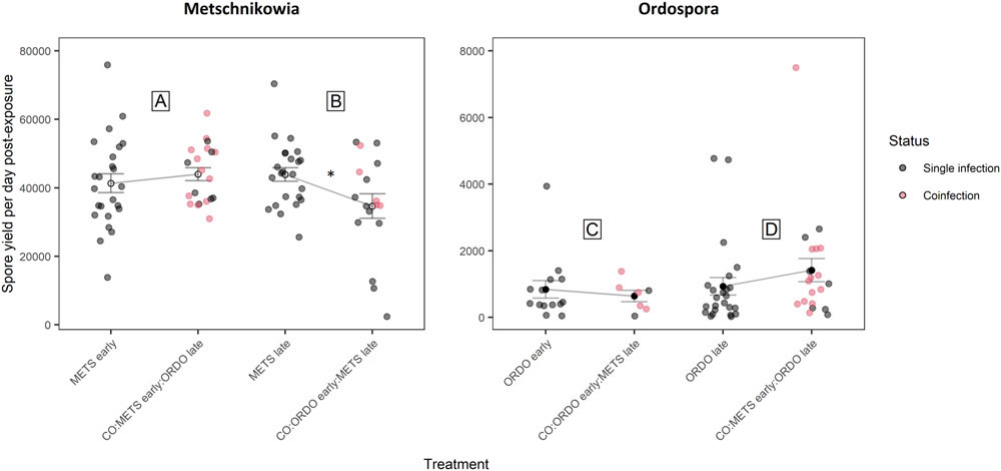
Graphical representation of parasite growth (computed as the ratio of spore yield upon host death and the number of days survived by the host, post-second exposure event) for the parasites *Metschnikowia* and *Ordospora*. Parasite growth was compared between single and co-exposure treatments, to answer the following: (A) How does *Metschnikowia* respond to later arrival of *Ordospora*? (B) How does *Metschnikowia* respond to prior infection by *Ordospora*? (C) How does *Ordospora* respond to later arrival of *Metschnikowia*? (D) How does *Ordospora* respond to prior infection by *Metschnikowia*? Coloured dots depict individuals which were confirmed to be coinfected by *Metschnikowia* and *Ordospora*. Error bars depict the standard error of the mean, which was computed by pooling singly and coinfected individuals in the co-exposure treatments. Significance levels are provided by analysis of variance (*F*-test) across single and co-exposure treatments with shared timing of infection: *P ⩽ 0.05.

**Fig. 5.**
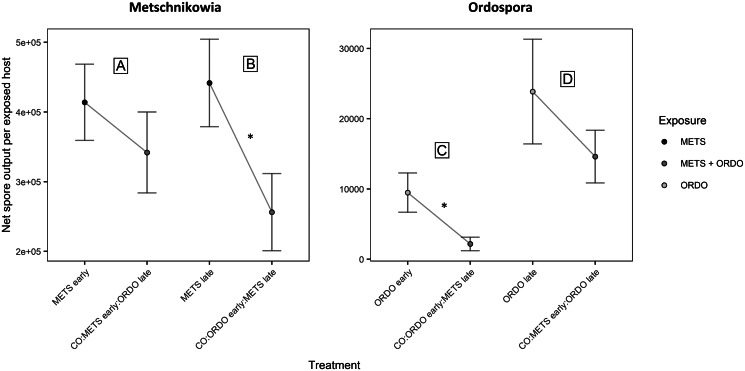
Graphical representation of the net spore output (per exposed host) for the parasites *Metschnikowia* and *Ordospora*, as compared between single and co-exposure treatments, to answer the following: (A) How does *Metschnikowia* respond to later arrival of *Ordospora*? (B) How does *Metschnikowia* respond to prior infection by *Ordospora*? (C) How does *Ordospora* respond to later arrival of *Metschnikowia*? (D) How does *Ordospora* respond to prior infection by *Metschnikowia*? Error bars depict the standard error of the mean. Significance levels are provided by analysis of variance (*F*-test) across single and co-exposure treatments with shared timing of infection: *P ⩽ 0.05.

Under prior arrival of *Metschnikowia*, the viability of experimental *Daphnia* did not differ between the single and co-exposure treatments, with 94.7% (METS early) and 89.7% (CO:METS early:ORDO late) of hosts surviving until day 9 post-exposure (Fig. 2A, Table 1). Among hosts considered viable, the probability of successful infection did not differ significantly between single (68.6%) and co-exposure (57.1%) treatments (Fig. 3A, Table 1). Parasite growth was comparable between single and co-exposure treatments (Fig. 4A, Table 1). Thus, the net output of *Metschnikowia* did not differ significantly across single and co-exposure treatments (Fig. 5A, Table 1).

**Table 1.** Analysis of variance (*F*-test or *χ*^2^ test) was performed across single and co-exposure treatments with shared timing of infection, to answer the following: (a) How does *Metschnikowia* respond to later arrival of *Ordospora*? (b) How does *Metschnikowia* respond to prior infection by *Ordospora*? (c) How does *Ordospora* respond to later arrival of *Metschnikowia*? (d) How does *Ordospora* respond to prior infection by *Metschnikowia*?

Response variable	Degree of freedom	*χ*^2^/*F* value	*P* value
(a) METS early | CO:METS early:ORDO late			
Host viability	75	0.6807	0.4093
Parasite infectivity	68	0.9820	0.3217
Parasite growth	42	0.5758	04522
Net output per exposed host	75	0.8109	0.3708
(b) METS late | CO:ORDO early:METS late			
Host viability	76	4.4597	**0**.**0347**
Parasite infectivity	53	0.0768	0.7817
Parasite growth	38	5.7688	**0**.**0213**
Net output per exposed host	76	0.4945	**0**.**0291**
(c) ORDO early | CO:ORDO early:METS late			
Host viability	78	3.8652	**0**.**0493**
Parasite infectivity	58	2.9877	0.0839
Parasite growth	19	0.1618	0.6920
Net output per exposed host	78	6.0996	**0**.**0157**
(d) ORDO late | CO:METS early:ORDO late			
Host viability	74	2.9155	0.0877
Parasite infectivity	70	1.2013	0.2731
Parasite growth	43	1.2613	0.2676
Net output per exposed host	73	1.2907	0.2596

A generalized linear model was used, assuming a binomial distribution of residuals for host viability of individual *Daphnia* (0 = early death, 1 = viable host) and infection status of individual *Daphnia* (0 = non infected, 1 = infected). A general linear model was used, assuming a normal distribution of residuals for parasite growth (rate of spore production per infected host) and the net spore output per exposed host. Significant *P* values (⩽0.05) are highlighted in bold.

#### How does *Metschnikowia* respond to prior infection by *Ordospora*?

Under late arrival of *Metschnikowia*, individuals which were first exposed to *Ordospora* suffered significant mortality during the early days of the experiment, with only 60.0% of hosts remaining viable (CO:ORDO early:METS late), as opposed to 81.6% in the single-exposure treatment (*METS late*) (Fig. 2B, Table 1). Infectivity did not differ significantly between the single (74.2%) and co-exposure (70.8%) treatments (Fig. 3B, Table 1). Parasite growth was significantly reduced in the co-exposure treatment (Fig. 4B, Table 1). Consequently, the net output of *Metschnikowia* in co-exposure was only half of that in the corresponding single-exposure treatment (Fig. 5B, Table 1).

#### How does *Ordospora* respond to later arrival of *Metschnikowia*?

Under prior arrival of *Ordospora*, the viability of experimental *Daphnia* was significantly reduced in the co-exposure treatment, with only 60.0% of hosts remaining viable (CO:ORDO early:METS late) compared to 80.0% in single exposure (ORDO early) (Fig. 2C, Table 1). There was a tendency towards higher infectivity in single exposure (43.8%) compared with the co-exposure treatment (29.2%) (Fig. 3C, Table 1). Parasite growth did not differ between the single and co-exposure treatments (Fig. 4C, Table 1). However, the net output of *Ordospora* was still 3-fold lower in co-exposure than in the single-exposure treatment (Fig. 5C, Table 1).

#### How does *Ordospora* respond to prior infection by *Metschnikowia*?

Under late arrival of *Ordospora*, there was a tendency towards higher viability in single exposure, with respectively 97.3% (ORDO late) and 87.2% (CO:METS early:ORDO late) of surviving hosts (Fig. 2D, Table 1). Infectivity did not differ between these treatments, with respectively 69.4% in single exposure and 61.8% in co-exposure (Fig. 3D, Table 1). Parasite growth did not differ either between those treatments (Fig. 4D, Table 1). Consequently, the net output of *Ordospora* did not differ significantly between single and co-exposure (Fig. 5D, Table 1).

### Host fitness

**Fig. 6.**
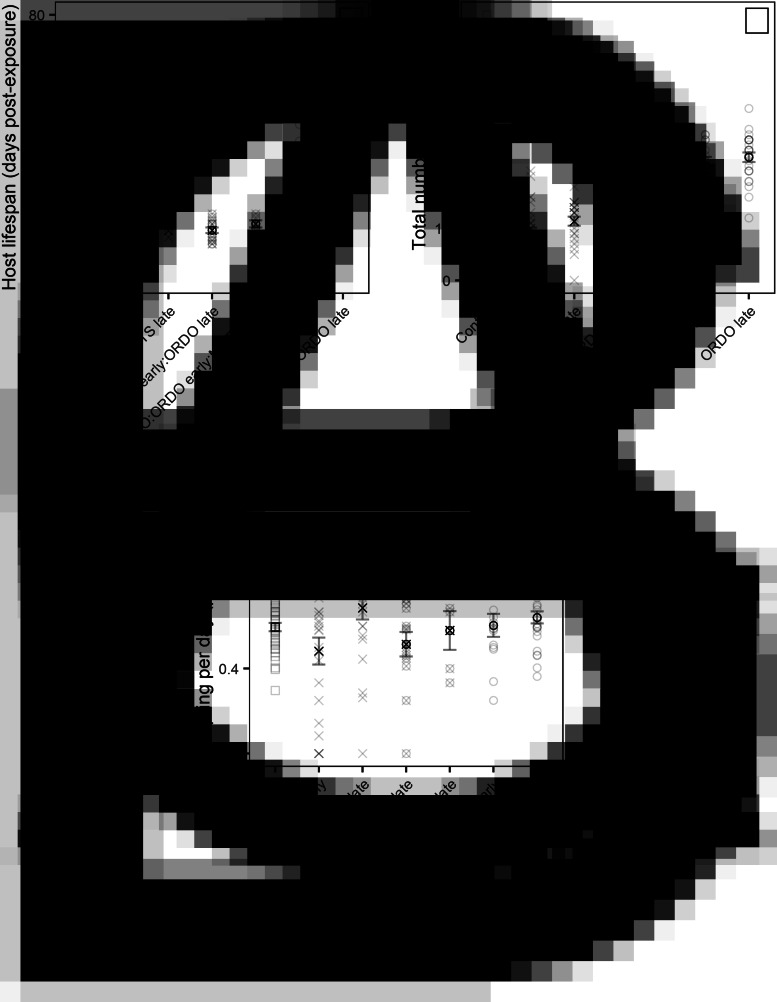
Graphical representation of (A) lifespan post-exposure, (B) total offspring production and the resulting (C) rate of offspring production (number of offspring per day post-exposure) compared for individual *Daphnia* across the control and all six exposure treatments. Only individuals successfully infected by one (single exposure) or both parasites (co-exposure) were included in the non-control treatments. Error bars depict the standard error of the mean.

Exposure had a significant effect on host lifespan (*F*_6,135_ *=* 138.4; *P* < 0.001) and total offspring production (*F*_6,135_ *=* 74.46; *P* < 0.001). On average, control *Daphnia* lived 56 days post-exposure (CI_95%_ ± 2.19; Fig. 6A) and produced 33 offspring (CI_95%_ ±2.28; Fig. 6B). In comparison, hosts singly infected by *Ordospora* lived 38 days post-exposure (±2.89; Fig. 6A) and produced 23 offspring (±1.62; Fig. 6B), while those singly infected by *Metschnikowia* lived 17 days (±1.54; Fig. 6A) and produced only ten offspring (±1.56; Fig. 6B). Single-exposure treatments with opposite timing of infection did not differ significantly from each other (Appendix, Table S3). The reduction in host lifespan and total offspring production induced by coinfection was comparable to that of single infections by *Metschnikowia*, but much stronger overall than the effect of single infections by *Ordospora*. Post-hoc analyses of the rate of offspring production indicate that such differences in fecundity were mostly driven by lifespan (Fig. 6C). While exposure had a significant effect on the rate of offspring production (*F*_6,135_ *=* 2.376; *P* *=* 0.033), the only significant pairwise comparison occurred between the METS early and METS late treatments, with the former reducing host fecundity to a greater extent (Tukey's HSD: *t*-value: 3.315, *P* = 0.018; Appendix, Table S3).

## Discussion

By exposing the host *D. magna* to sequential infections of the gut-dwelling microsporidium, *O. colligata* and the haemolymph-infecting yeast, *M. bicuspidata*, we investigated the potential for priority effects at the within-host level, in a system of sympatric species. We simulated two orders of arrival, designed to reflect contrasting patterns of parasite succession. In sequential exposures where *Metschnikowia* arrived first (CO:METS early:ORDO late), parasite transmission traits (parasite infectivity, parasite growth) did not differ significantly from single exposures. However, in sequential exposures where *Ordospora* arrived first (CO:ORDO early:METS late), parasite growth was reduced for the fungal parasite. Though infectivity was not significantly impacted, there was also higher host mortality in this treatment, which contributed to a decrease in the net spore output of both parasites (i.e. a comprehensive measure of parasite fitness).

### Performance of *Metschnikowia* under single vs sequential infections

Under prior residency of *Metschnikowia*, sequential exposures were not shown to influence its transmission potential, as none of the recorded parameters differed between single exposure (METS early) and co-exposure (CO:METS early:ORDO late). This apparent lack of effect was unexpected, as it somewhat contradicts previous findings involving this parasite. When pitting *Metschnikowia* against the ichthyosporean gut parasite *Caullerya mesnili*, Lohr *et al*. (2010*b*) found that given prior residency, *Metschnikowia* took longer to develop, and produced fewer spores in coinfection. Similarly, Clay *et al*. (2019) observed lower production of fungal spores in coinfected hosts, when *Metschnikowia* was first to arrive against the bacterium *Pasteuria ramosa*, as opposed to the treatment where it arrived second. Both studies suggest that *Metschnikowia* generally does not fare well under prior residency. However, the authors co-exposed *Daphnia* hosts to parasites that are considerably more virulent than *Ordospora*. Both *C. mesnili* and *P. ramosa* are known to induce complete castration of their hosts (Bittner *et al*., 2002; Ebert *et al*., 2004; Jensen *et al*., 2006; Lohr *et al*., 2010*a*). Parasites that shut down reproduction entirely (i.e. parasitic castration) are thought to redirect considerable amount or resources, that would normally support reproductive effort of the host, towards increased growth or survivorship instead (Baudoin, 1975). This difference in exploitation strategy may partly explain why *Metschnikowia* would experience strong priority effects against such virulent parasites, while demonstrating no apparent response to the later establishment of *Ordospora*.

By contrast, we found evidence for reduced transmission of *Metschnikowia*, when it was preceded by the gut parasite. Sequential exposure resulted in a 2-fold reduction of *Metschnikowia*'s net spore output, which was seemingly driven by two parameters of parasite fitness. First, parasite growth of *Metschnikowia* was slightly reduced in sequential exposure (CO:ORDO early:METS late), as opposed to the single-exposure treatment (METS late). This effect may be attributed to prior resource sequestration by the gut parasite. Intracellular microsporidian parasites ensure within-host growth by scavenging ATP molecules from host cells, through the activity of nucleotide transporters (Tsaousis *et al*., 2008; Smith, 2009) and further interactions with host mitochondria (Terry *et al*., 1997). Considering that infection by *Ordospora* takes place in the gut epithelium, prior sequestration of resources at the interface between the gut lumen and the haemolymph (i.e. where *Metschnikowia* completes its development and reproduction cycle) seems plausible. Second, a significant reduction of host viability was recorded in hosts that were first exposed to *Ordospora*, prior to *Metschnikowia* (CO:ORDO early:METS late), which resulted in a large proportion of co-exposed hosts not progressing towards successful reproduction of *Metschnikowia*.

While the mechanism responsible for such high mortality is difficult to infer from our results, this pattern is reminiscent of the ultrainfection phenomenon first described by Sofonea *et al*. (2015). Ultrainfection occurs when two parasites display adaptive levels of virulence and growth in single infection, while double infection triggers ‘explosive’ levels of host mortality, that are normally not found in each respective species. For this reason, coinfections are often hidden in the population, as cases that do occur only exist for a brief span of time, quickly interrupted by host death (Sofonea *et al*., 2017). With regards to the present study, the CO:ORDO early:METS late treatment did result in excessive host mortality, which also contributed to a very low number of successfully coinfected hosts. A similar phenomenon has been described in nature, where interspecific coinfection of an insect host generates lethal levels of damage from a viral pathogen that is otherwise considered avirulent (Nazzi *et al*., 2012).

Additionally, it has been observed that prior infection by a gut parasite can modify the structural integrity of the gut in *Daphnia*, which in turn modulates the probability of fungal spores successfully crossing into the haemolymph (T. Stewart Merrill, personal communication). Thus, we suspected prior colonization of epithelial cells by *Ordospora* could have altered susceptibility to *Metschnikowia*; however, parasite infectivity did not differ from single exposure in this treatment.

### Performance of *Ordospora* under single vs sequential infections

In single-exposure treatments, the overall infection success of *Ordospora* was lower when it was inoculated on day 5. Although we suspect possible heterogeneity between spore solutions may have contributed to this observation (as different parasite inoculates were used on days 5 and 7), age and body size-related effects could have further influenced infectivity (Izhar and Ben-Ami, 2015; Garbutt and Little, 2017). For instance, filtering rate and permeability of the gut epithelium (i.e. thickness of cell wall) in *Daphnia* have been shown to directly correlate with age and size class (Burns, 1969; Stewart Merrill *et al*., 2019). As *D. magna* can reach maturity starting from 7 days at 20°C (Lampert, 1993), the initial exposure of pre-adults *Daphnia* (i.e. from days 5–7) as opposed to potentially mature individuals (i.e. from days 7–9) may have influenced the parasite's infection success (Ben-Ami, 2019).

Independent of this observation, sequential exposure reduced transmission of *Ordospora*, when it was first to infect the host (CO:ORDO early:METS late). Contrary to our observations on *Metschnikowia*, these results seem to have been driven mostly by increased mortality of co-exposed hosts, as parasite growth did not differ between the single and co-exposure treatments. While our method for quantifying spores did not allow us to monitor the continuous shedding of propagules from live hosts, the number of spore clusters recorded in the gut of infected individuals increases exponentially throughout the course of infection (Mangin *et al*., 1995; Kirk *et al*., 2018), with each cluster bearing up to 60 infective stages (Kirk *et al*., 2019). This suggests that spore yield recorded upon fixation of the host can be used to approximate the parasite's progression along the gut epithelium (i.e. infection intensity) and overall reproductive success. Although previous coinfection experiments using *Ordospora* were not available for comparison, *C. mesnili* benefited from an increase in spore production, when it was first to arrive in coinfection with *Metschnikowia* (Lohr *et al*., 2010*b*). As mentioned above, the contrasting priority effects observed here may stem from distinct strategies of host exploitation and varying degrees of fitness impairment, as *Ordospora* is one of the least virulent endoparasites commonly found in *Daphnia* (Ebert, 2005).

Due to external factors, such as selective predation on infected individuals (Duffy *et al*., 2005; Johnson *et al*., 2006; Goren and Ben-Ami, 2017), *Daphnia* in their natural habitat may not experience such long lifespans as those observed in controlled conditions (instead, rarely surviving beyond 20 days; Lampert, 1993). In the present study, individuals which were successfully coinfected by both parasites experienced similar lifespan as those singly infected by *Metschnikowia*, but lived only half the span of those singly infected by *Ordospora* (Fig. 6A). Therefore, coinfections in nature may contribute fewer infective propagules to the overall transmission of *Ordospora*, especially when no benefit to coinfection was observed, that would help compensate this reduction in host lifespan.

### From parasite phenology to sequential exposure

The phenology of symbionts often varies, causing them to emerge among a host population sequentially (Schmidt *et al*., 2007; Dumbrell *et al*., 2011). Because the probability of being the first to infect directly correlates with a parasite's prior prevalence in the population (Clay *et al*., 2018), differences in species emergence patterns may in turn facilitate the occurrence of priority effects at the within-host level. While *Ordospora* may reach very high prevalence in natural populations of *D. magna* (Ebert, 2001), reportedly nearing 40% in shallow eutrophic ponds (Decaestecker *et al*., 2005), much lower prevalences have been recorded for *Metschnikowia* in similar environments (<10%, Stirnadel and Ebert, 1997). Thus, co-occurrence of these two species could imply that a significant proportion of the host population may have already encountered *Ordospora*, around the time when *Metschnikowia* increases to peak prevalence (i.e. in the late summer).

Additionally, spores of these two parasites are likely to be found in separate locations of the water column. While epidemics of *Ordospora* typically start from infectious spore banks, following a period of inactivity from host populations (Mangin *et al*., 1995), subsequent infections are likely to result in the continuous shedding of spores from live hosts. Because infective stages are able to disperse in the water (Mangin *et al*., 1995; Kirk *et al*., 2018), these may be encountered as free-floating spores across the upper parts of the water column. By contrast, spores of *Metschnikowia* gradually build up in the sediment, where infected hosts sink to and decompose after succumbing to infection (Duffy and Hunsberger, 2019). However, selective predation of spore-bearing individuals may contribute to the occasional resuspension of the parasite in the water column, as non-damaged asci can remain infectious following their passage through a fish's digestive tract (Duffy, 2009). Due to particularly strong diel vertical migration behaviour in *D. magna* (De Meester, 1992), this species is especially prone to contamination from infectious spore banks (Decaestecker *et al*., 2002, 2004). However, differences in the likelihood of spore encounter may also be driven by individual variability in phototactic behaviour, which exhibits strong genotypic variation among clones of *D. magna* (De Meester, 1989; De Meester *et al*., 1994). For instance, positively phototactic genotypes may recruit a higher proportion of free-floating microsporidian spores during the day, while being exposed to buried spore banks during the night. Finally, it has been shown that *D. magna* individuals infected with *Ordospora* exhibit much deeper position than uninfected ones in artificial mesocosms (Fels *et al*., 2004). This suggests that prior infection by *Ordospora* may also influence host behaviour in such a way that secondary infections (e.g. by *Metschnikowia*) are facilitated in nature.

Within-host interactions between symbionts may scale up to influence host-parasite dynamics at the community level (Mordecai *et al*., 2016; Marchetto and Power, 2018; Karvonen *et al*., 2019), a phenomenon that has been demonstrated experimentally (Halliday *et al*., 2017). For instance, mechanisms of positive or negative frequency dependence may arise from system-specific priority effects (Clay *et al*., 2018). The unilateral priority effects highlighted in this study (i.e. reduced transmission under prior arrival of *Ordospora*) are likely to occur in populations where both parasites are sympatric. These may be of particular importance during the early phase of parasite emergence, when every successful infection helps to kick-start a parasite's successful outbreak in the environment. For instance, species that usually emerge later in the season (e.g. *Metschnikowia*) are effectively starting in an environment where most – if not all – available hosts may have previously encountered a competing parasite species (e.g. *Ordospora*). Parasites that tend to suffer from late residency might face a ‘critical early point’ in their epidemic curve, during which most infections with previously infected hosts could result in a suboptimal outcome, potentially slowing – if not preventing – their successful establishment and emergence in the environment.

## Concluding remarks

Our results suggest that specific patterns of parasite succession, with prior emergence of the microsporidium *Ordospora* over the yeast *Metschnikowia* (i.e. a plausible scenario in natural populations) may limit the transmission of both species, due to (i) impaired spore production of the yeast and (ii) maladaptive levels of host mortality that are not found in single infections. We also highlight the inherent specificity of priority effects among common parasites of *Daphnia*, showing that contrasting responses to sequential infections can be observed across a microsporidian gut parasite and functionally similar species. Thus, we encourage further research to consider other assemblages of ecologically relevant parasites, while monitoring temporal succession patterns that are observed in the field. Changes in parasite phenology could be especially relevant in light of climate change: distinct species may react differently to specific environmental triggers – such as light, temperature or nutrient availability – that are known to stimulate the emergence of resting stages, transmission and within-host reproduction (e.g. Overholt *et al*., 2012; Kirk *et al*., 2018). Elevated freshwater temperatures may cause asymmetric shifts between the overlapping epidemic curves of waterborne parasites, which could have implications for the likelihood of sequential infections at the within-host level.

## Data Availability

The data supporting this study can be found at https://doi.org/10.5281/zenodo.5223097

## Supplementary material

For supplementary material accompanying this paper visit https://doi.org/10.1017/S0031182021001384. click here to view supplementary material
