# Supplementary material for: Sequential infection of Daphnia magna by a gut microsporidium followed by a haemolymph yeast decreases transmission of both parasites
Source: Parasitology. 2021 Aug 10;148(13):1566–77. doi: 10.1017/S0031182021001384 (PMC8564772; doi:10.1017/S0031182021001384)
Supplement: Supplementary file 1 [file S0031182021001384sup001.docx]

**Appendix**

**Table S1:** Proportion of *viable hosts*: experimental *Daphnia* which survived until at least day 9 post-exposure (i.e., earliest observation of infection symptoms for *Metschnikowia*) or day 11 post-exposure (i.e., earliest observation of infection symptoms for *Ordospora*); proportion of *infected* hosts: experimental *Daphnia* confirmed to have produced spores of the parasites (a) *Metschnikowia* or (b) *Ordospora*. Individuals which did not survive until at least both inoculation events had occurred (beyond experimental day 7) were excluded; reported proportions are computed from ≤ 40 surviving hosts per exposure treatment.

1. **Successful infections by *Metschnikowia***

| **Exposure** | **Proportion of viable hosts** | | **Proportion of infected hosts** | |
| --- | --- | --- | --- | --- |
| METS early | 36/38 | 0.947 | 24/36 | 0.686 |
| CO:METS early:ORDO late | 35/39 | 0.897 | 20/35 | 0.571 |
| METS late | 31/38 | 0.816 | 23/31 | 0.742 |
| CO:ORDO early:METS late | 24/40 | 0.600 | 17/24 | 0.708 |
| Total (≤ 160 replicates) | 126/155 | 0.813 | 84/126 | 0.666 |

1. **Successful infections by *Ordospora***

| **Exposure** | **Proportion of viable hosts** | | **Proportion of infected hosts** | |
| --- | --- | --- | --- | --- |
| ORDO early | 32/40 | 0.800 | 14/32 | 0.438 |
| CO:ORDO early:METS late | 24/40 | 0.600 | 7/24 | 0.292 |
| ORDO late | 36/37 | 0.973 | 25/36 | 0.694 |
| CO:METS early:ORDO late | 34/39 | 0.872 | 21/34 | 0.618 |
| Total (≤ 160 replicates) | 126/156 | 0.808 | 67/126 | 0.532 |

**Table S2:** Logistic regression performed across combinations of single exposure and co-exposure treatments with the same timing of infection. The number of individuals surviving until the first possible detection of the parasites *Metschnikowia* (i.e., from day 9 onward) or *Ordospora* (i.e., from day 11 onward) was compared to baseline mortality in the control treatment. A generalized linear model was used, assuming a binomial distribution of residuals. Significant *P*-values (≤ 0.05) are highlighted in bold.

| **Response variable** | **Degree of freedom** | **χ^2^** | ***P*-value** |
| --- | --- | --- | --- |
| Control \| METS early \| CO:METS early:ORDO late | | | |
| Host viability (≥ day 9) | 2; 113 | 5.762 | 0.056 |
| Control \| METS late \| CO:ORDO early:METS late | | | |
| Host viability (≥ day 9) | 2; 114 | 25.829 | **< 0.001** |
| Control \| ORDO early \| CO:ORDO early:METS late | | | |
| Host viability (≥ day 11) | 2; 116 | 27.921 | **< 0.001** |
| Control \| ORDO late \| CO:METS early:ORDO late | | | |
| Host viability (≥ day 11) | 2; 112 | 8.9352 | **0.0115** |

**Table S3:** Results of Tukey’s HSD test applied to a one-way analysis of variance (ANOVA), with (a) host lifespan, (b) total offspring production and (c) the rate of offspring production as response variables, compared across the control and all exposure treatments. Only significant *P*-values (≤ 0.05) are reported and highlighted in bold.

1. **Host lifespan**

| **Compared levels** | | **Estimate** | **Standard error** | **t value** | ***P*-value** |
| --- | --- | --- | --- | --- | --- |
| Control | METS early | +39.33 | 1.80 | 21.835 | **< 0.0001** |
| Control | METS late | +39.97 | 1.83 | 21.900 | **< 0.0001** |
| Control | ORDO late | +18.59 | 1.78 | 10.448 | **< 0.0001** |
| Control | ORDO early | +19.20 | 2.16 | 8.889 | **< 0.0001** |
| Control | CO:METS early:ORDO late | +41.56 | 2.22 | 18.716 | **< 0.0001** |
| Control | CO:ORDO early:METS late | +39.63 | 3.29 | 12.056 | **< 0.0001** |
| ORDO early | METS early | +20.14 | 2.32 | 8.665 | **< 0.0001** |
| ORDO early | METS late | +20.78 | 2.34 | 8.870 | **< 0.0001** |
| ORDO early | CO:METS early:ORDO late | +22.35 | 2.66 | 8.398 | **< 0.0001** |
| ORDO early | CO:ORDO early:METS late | +20.43 | 3.60 | 5.674 | **< 0.0001** |
| ORDO late | METS early | +20.75 | 1.97 | 10.507 | **< 0.0001** |
| ORDO late | METS late | +21.39 | 2.00 | 10.712 | **< 0.0001** |
| ORDO late | CO:METS early:ORDO late | +22.96 | 2.36 | 9.718 | **< 0.0001** |
| ORDO late | CO:ORDO early:METS late | +21.04 | 3.39 | 6.215 | **< 0.0001** |

1. **Total offspring production**

| **Compared levels** | | **Estimate** | **Standard error** | **t value** | ***p*-value** |
| --- | --- | --- | --- | --- | --- |
| Control | METS early | +24.64 | 1.48 | 16.614 | **< 0.001** |
| Control | METS late | +22.13 | 1.50 | 14.724 | **< 0.001** |
| Control | ORDO early | +11.40 | 1.79 | 6.412 | **< 0.001** |
| Control | ORDO late | +9.83 | 1.46 | 6.713 | **< 0.001** |
| Control | CO:METS early:ORDO late | +25.70 | 1.82 | 14.064 | **< 0.001** |
| Control | CO:ORDO early:METS late | +23.67 | 2.71 | 8.749 | **< 0.001** |
| ORDO early | METS early | +13.24 | 1.91 | 6.921 | **< 0.001** |
| ORDO early | METS late | +10.72 | 1.92 | 5.562 | **< 0.001** |
| ORDO early | CO:METS early:ORDO late | +14.302 | 2.19 | 6.528 | **< 0.001** |
| ORDO early | CO:ORDO early:METS late | +12.271 | 2.96 | 4.141 | **0.001** |
| ORDO late | METS early | +14.807 | 1.63 | 9.109 | **< 0.001** |
| ORDO late | METS late | +12.292 | 1.64 | 7.480 | **< 0.001** |
| ORDO late | CO:METS early:ORDO late | +15.871 | 1.95 | 8.160 | **< 0.001** |
| ORDO late | CO:ORDO early:METS late | +13.840 | 2.786 | 4.967 | **< 0.001** |

1. **Rate of offspring production**

| **Compared levels** | | **Estimate** | **Standard error** | **t value** | ***p*-value** |
| --- | --- | --- | --- | --- | --- |
| METS late | METS early | +0.20 | 0.06 | 3.315 | **0.0185** |
